# Drug Absorption Efficiency in *Caenorhbditis elegans* Delivered by Different Methods

**DOI:** 10.1371/journal.pone.0056877

**Published:** 2013-02-25

**Authors:** Shan-Qing Zheng, Ai-Jun Ding, Guo-Ping Li, Gui-Sheng Wu, Huai-Rong Luo

**Affiliations:** 1 State Key Laboratory of Phytochemistry and Plant Resources in West China, Kunming Institute of Botany, Chinese Academy of Sciences, Kunming, Yunnan, China; 2 University of Chinese Academy of Sciences, Beijing, China; 3 The key laboratory of Geriatrics, Beijing Hospital & Beijing Institute of Geriatrics, Ministry of Health, Beijing, China; 4 Key Laboratory of Animal Models and Human Disease Mechanisms, Chinese Academy of Sciences & Yunnan Province, Kunming Institute of Zoology, Kunming, Yunnan, China; CNRS, France

## Abstract

**Background:**

*Caenorhbditis elegans* has being vigorously used as a model organism in many research fields and often accompanied by administrating with various drugs. The methods of delivering drugs to worms are varied from one study to another, which make difficult in comparing results between studies.

**Methodology/Principal Findings:**

We evaluated the drug absorption efficiency in *C. elegans* using five frequently used methods with resveratrol with low aqueous solubility and water-soluble 5-Fluoro-2′-deoxyuridine (FUDR) as positive compounds. The drugs were either applied to the LB medium with bacteria OP50, before spreading onto Nematode Growth Medium (NGM) plates (LB medium method), or to the NGM with live (NGM live method) or dead bacteria (NGM dead method), or spotting the drug solution to the surface of plates directly (spot dead method), or growing the worms in liquid medium (liquid growing method). The concentration of resveratrol and FUDR increased gradually within *C. elegans* and reached the highest during 12 hours to one day and then decreased slowly. At the same time point, the higher the drug concentration, the higher the metabolism rate. The drug concentrations in worms fed with dead bacteria were higher than with live bacteria at the same time point. Consistently, the drug concentration in medium with live bacteria decreased much faster than in medium with dead bacteria, reach to about half of the original concentration within 12 hours.

**Conclusion:**

Resveratrol with low aqueous solubility and water-soluble FUDR have the same absorption and metabolism pattern. The drug metabolism rate in worms was both dosage and time dependent. NGM dead method and liquid growing method achieved the best absorption efficiency in worms. The drug concentration within worms was comparable with that in mice, providing a bridge for dose translation from worms to mammals.

## Introduction

Since *Caenorhabditis elegans* (*C. elegans*) was chosen as a model organism to study genetics [1], the worms have been widely used in many research areas, either as research or drug screening model [2], [3], such as in development [4]–[6], lipid metabolism and obesity [7]–[9], aging [10], [11], neurodegenerative disease [12]–[14], antimicrobials [15]–[17], virulence and parasites [18], biomedical and environmental toxicology [19], and cancer [20]. Treating worms with tool drugs was frequently used in these studies. For the tiny nature of the worms, the drug delivery methods were indirect and often varied from one study to another. The drugs were either applied to the LB medium growing the bacteria, the food of worms (LB medium method), or directly spotted onto the surface of NGM plates (spot dead method), or to the NGM with live (NGM live method) [21] or dead bacteria (NGM dead method) spread on the surface of the plates [22]–[24]. Another method was to keep the worms in liquid medium (liquid growing method) [15], [25], [26]. These different delivery methods might result in different drug absorption efficiency, causing confusing results between different studies [25]–[28]. Moreover, the resistance of *C. elegans* to pharmacological perturbation appeal an effective drug delivery approach to make *C. elegans* as a screening tool for novel small bioactive molecules [29].

Currently, the information for drug absorption in worms was scarce. To bridge this gap, we evaluated the drug absorption efficiency in worms of five frequently used drug-delivering approaches with the test compounds resveratrol and 5-Fluoro-2′-deoxyuridine (FUDR). Our data indicated that resveratrol and FUDR administrated with NGM dead method and liquid growing method achieved the best absorption efficiency in worms, while the spot dead method was the economic approach.

## Materials and Methods

### Drug administration, worm culturing and harvesting

**Figure 1:**
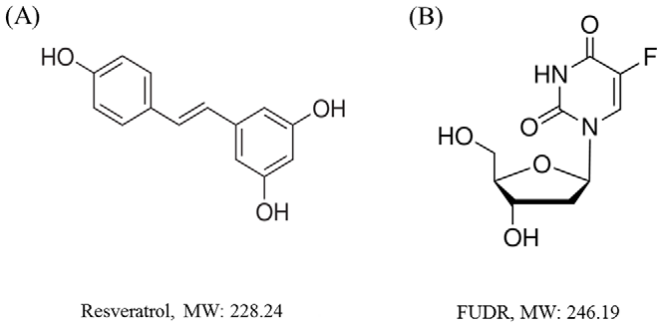
The structure and molecular weight of resveratrol (A) and FUDR (B).

The wild type N2 *C. elegans* was provided by the *Caenorhabditis Genetics Center* (CGC). Resveratrol and FUDR (Sigma Aldrich) were used as the test compounds. Resveratrol (3,5,4′-trihydroxy-trans-stilbene) is a type of natural phenol with low aqueous solubility and the molecular weight of 228.24 (Figure 1A). Resveratrol has been shown to extend the lifespan of yeast, fly and *C. elegans* with clear mechanism of action [30]–[33]. FUDR, with the molecular weight of 246.2 (Figure 1B), was soluble in water at the concentration to 50 mg/mL. FUDR was widely used to inhibit the worms to lay eggs in research with the concentration of FUDR from 40 µM to 50 µM [34], [35].

**Figure 2:**
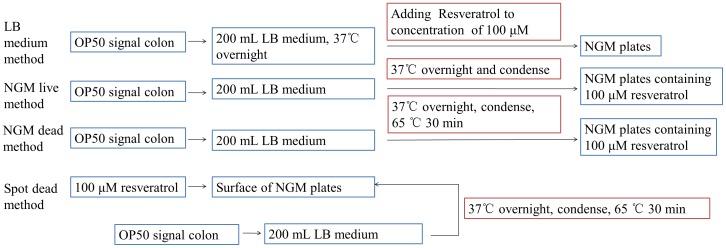
The summary of drug administration for LB medium method, NGM live method, NGM dead method, and spot dead method.

Resveratrol was dissolved in DMSO to 100 mM as stock solution. In LB medium method, the resveratrol stock solution was diluted into LB liquid medium containing OP50 *E. coli* bacteria to a final concentration of 100 µM and 1.5 mL of the bacteria solution was applied to each NGM plate (100 mm diameter). In spot dead method, 1.5 mL of 100 µM resveratrol was spotted onto the surface of the NGM plate, then covered with dead bacteria. In NGM live method, the resveratrol stock solution was diluted with NGM (below 65°C after boiled) to the concentration of 100 µM. Then the NGM was poured into petri plates as supporting bed for worms and live OP50 was applied to the surface of NGM plates. In NGM dead method, the plates were made as the NGM live method, except that the food OP50 bacteria was killed by incubating in 65°C for 30 minutes [24]. The drug administration of above four methods was summarized in Figure 2. The maintenance of *C. elegans* in liquid medium was described as previously [23] with slight modification. Briefly, the synchronized N2 adult day 1 worms were cultured in 50 mL centrifuge tubes that contained 35 mL S medium [23], the concentration of resveratrol in S medium was 100 µM. Dead bacteria were added to S medium as food. FUDR was dissolved in H_2_O to 50 mM as stock solution. The procedures of treatment of worms with FUDR were the same with resveratrol, except that the final concentration of FUDR in the five treatment methods was 50 µM.

About 5,000–10,000 adult day 1 wild type worms were transferred to each NGM plate (100 mm diameter). For the liquid growing method, the worms were cultured in several 50 mL centrifuge tubes with each containing about 35 mL S medium [23]. Worms in each method were harvested by using cold M9 buffer at the 10 min, 30 min, 1 hr, 3 hr, 6 hr, 12 hr, day 1, day 2, day 4, day 7, day 14 and day 20 after treated with the compounds [23], and collected to 15 mL centrifuge tubes. The control group without treatment with compound was also harvested. The tubes were putted into ice for 10 minutes, then spin for 2 minutes at 1,150× g to precipitate the worms. The worm pellets were rinsed three times with cold M9 buffer, air dry, and weighed. The worm pellets were resuspended by using 1 mL methyl ethanol (HPLC grade) (for resveratrol) or H_2_O (for FUDR) and sonicated 50 times (200 V, operation 5 seconds every 5 seconds). Then, the worm solution was centrifuged under 12, 000× g for 3 minutes. The supernatant of the worm solution containing resveratrol or FUDR was transferred to a 1.5 mL centrifuge tube.

To test the drug metabolism, the worms treated with 400 µM, 200 µM, 100 µM, 50 µM, 25 µM, and 12.5 µM of resveratrol and FUDR, respectively, for 6 hours under NGM dead method were transferred to NGM plates without resveratrol and FUDR. Then, the worms were harvested at the 10 min, 30 min, 1 hr, 2 hr, 3 hr, 4 hr, 6 hr, 8 hr, 12 hr, and 16 hr time points. Subsequent sample preparation was the same as described above.

### Measurement of the concentration of resveratrol and FUDR using HPLC

The Agilent 1200 with auto-injector and dual absorbance UV detector was used for sample analysis. All samples including standard resveratrol solutions were filtered with 0.45-µm organic filter. Then, 10 µL of each sample was injected into the system and separated in a reversed-phase Agilent C18 column (ZORBAX, SB-C18, 4.6×250 mm, 5 µM) containing 27% of C_2_H_3_N and 73% of H_2_O at mobile phase, with flow rate of 1 mL/min and temperature at 30°C, and detected at 303 nm wavelength. For FUDR, the solutions passing through the mobile phase with flow rate of 1 mL/min at 20°C were 10%–12% of MeOH in H_2_O for over 5 min, followed by 12%–15% of MeOH in H_2_O for 5 min, finally with 50% of MeOH for 3 min. FUDR was detected at 268 nM wavelength.

To calculate the concentration of resveratrol and FUDR in worms, the standard concentration curve of resveratrol and FUDR were made first. A series of standard solution of resveratrol were made freshly before analysis by diluting the methyl alcohol solution of resveratrol from stock solution of 2 g/L to working standard solutions of 0.5, 20, 80, 500, 600, 800, 1,200 mg/L, respectively. FUDR was diluted with H_2_O to 1, 5, 20, 100, 300, 800 mg/L, respectively. Calibration curve was plotted with the standard concentrations as the x-axis and the detected peak area signals as y-axis. The parameters of slope, intercept and correlation coefficient were carried out by linear regression.

The detection limits were evaluated according to the criteria that the signal to noise ratio should be >3. The precision was evaluated by the relative standard deviation (RSD) of the recovery test. To carry out the recovery tests, the standard samples of drugs with three known concentrations were spiked into the blank worm samples, subsequent drug isolation procedure and detection conditions were the same as other test samples. The recovery rate was indicated by the ratio of the detected concentration of the standard drugs to the spiked concentration. The intra-day precision was evaluated by conducting three times of the recovery test at different time point within one day. The inter-day precision was evaluated by conducting three times of the recovery tests on each day, with total of three days within one week. The repeatability and stability of drug absorption efficiency in worms was also analyzed by repeating the NGM dead method for six times.

## Results

### Validation of HPLC method to detecting the concentration of resveratrol and FUDR in worms

The retention time of the standard resveratrol and FUDR was 7.2 and 5.2 minutes, respectively. The regression equation of the standard curve for resveratrol and FUDR were y = 0.029x+0.517 and y = 12.131x−29.491, respectively. The parameters of the linear curve were carried out by analyzing three independent experiments with the determination co-efficiency of r^2^ = 0.999 for resveratrol and r^2^ = 0.9995 for FUDR. This calibration curve showed an excellent linearity in the concentration range of 20–1,200 mg/L for resveratrol and of 1–800 mg/L for FUDR.

**Figure 3:**
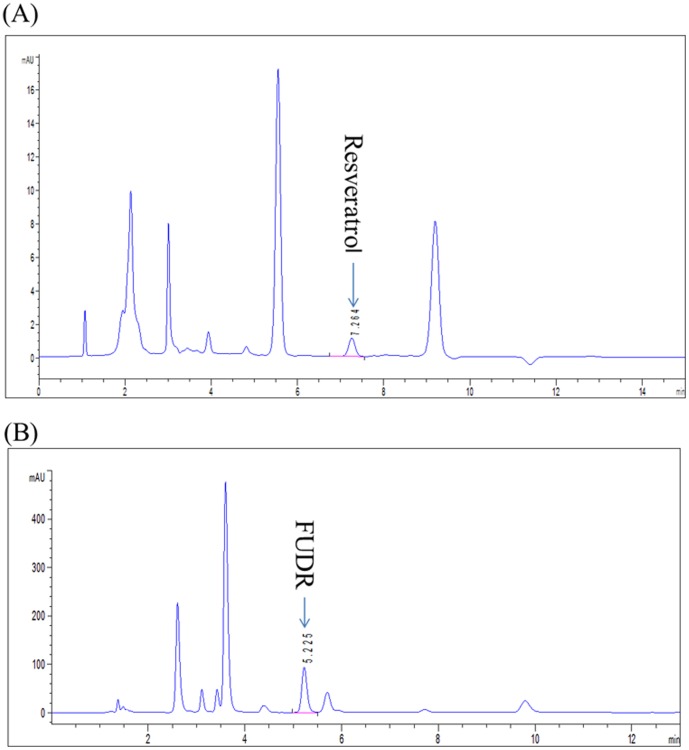
The HPLC profile and retention time of resveratrol and FUDR extracted from the day 1 worms. The retention time of resveratrol (A) and FUDR (B) in the worms was 7.2 min and 5.2 min, respectively.

The relative standard deviation (RSD) of the six repeated experiments of resveratrol and FUDR absorption efficiency of NGM dead method was 3.8% and 2.5%, respectively (Figure 3, Table 1). The recovery rate in the recovery test of resveratrol standard samples with concentration of 35, 60, and 100 mg/L were 100.6%, 98.6%, and 101.0%, respectively. The relative standard deviation for intra-day and inter-day in the recovery test of resveratrol standard samples with concentration of 35, 60, 100 mg/L were 2.99 and 3.76, 1.68 and 1.21, 1.42 and 1.49, respectively (RSD<3.8%, Table 2). The recovery rate in the recovery test of FUDR standard samples with concentration of 20 and 35 mg/L were 99.4% and 99.8%, respectively. The relative standard deviation for intra-day and inter-day in the recovery test of FUDR standard samples with concentration of 20 and 35 mg/L were 0.92 and 1.02, 0.73 and 0.85, respectively (RSD<1.1%, Table 2).

**Table 1:** The repeatability and stability of the HPLC method.

		Resveratrol		FUDR	
	Injected volume (µL)	Worm weight (mg)	Peak area (mAU*s)	Worm weight (mg)	Peak area (mAU*s)
**Experiment 1**	10	150	3.32298	30	34.2
**Experiment 2**	10	150	3.25668	30	36.4685
**Experiment 3**	10	150	3.32298	30	34.2
**Experiment 4**	10	150	3.36413	30	34.7917
**Experiment 5**	10	150	3.55011	30	34.5687
**Experiment 6**	10	150	3.56435	30	34.2349
**Average**			3.39687		34.7439
**Standard Deviation**			0.128978		0.878403
**Relative Standard Deviation (%)**			3.8		2.5

The day 1 worms cultured by using the NGM dead method were used in these tests. The table showed the six individual experiments.

**Table 2:** The precision of the HPLC detection of resveratrol and FUDR.

	Standard	Detected	Recovery rate	Relative Standard Deviation (%)	
	(mg/L)	(mg/L) ±SD	(%)	Intra-day	Inter-day
**Resveratrol**	35	35.2±1.05	100.6	2.99	3.76
	60	59.2±1.00	98.6	1.68	1.21
	100	101.0±1.44	101	1.42	1.49
**FUDR**	20	19.88±0.18	99.4	0.92	1.02
	35	34.92±0.26	99.8	0.73	0.85

The precision was evaluated through the relative standard deviation (RSD) of the recovery test by determining the recovery signal of the standard samples with three different concentrations each mixed into one of control samples.

### Drug absorption efficiency of the five drug delivering methods

**Figure 4:**
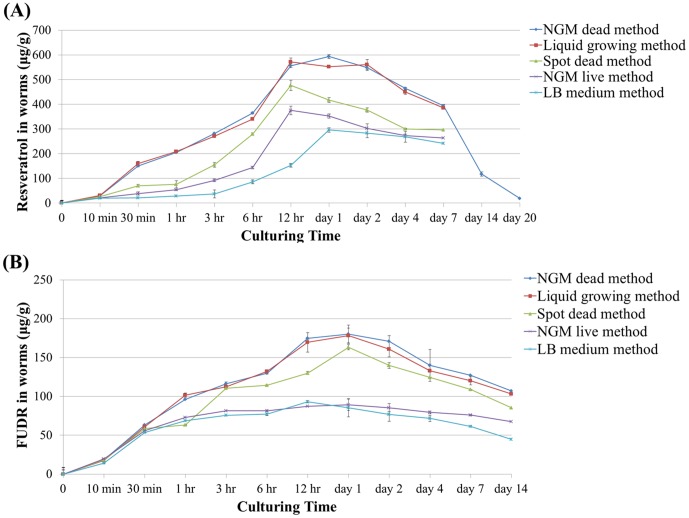
The drug absorption efficiency of worms administrated with 100 µM resveratrol (A) or 50 µM FUDR (B) by five delivering methods (µg/g). The concentration of drugs in worms was presented as µg/g. The figure showed the average of three repeated experiments for each method. The details of data were summarized in Table S1 and S2.

We tested the concentration of resveratrol and FUDR in worms administrated with drugs by five delivery methods at the time point of 10 min, 30 min, 1 hr, 3 hr, 6 hr, 12 hr, day 1, day 2, day 4, day 7, day 14, and day 20. The concentration of resveratrol and FUDR in worms accumulated steadily as time passing on and reached the highest at day one, then decreased gradually (Figure 4, Tables S1 and S2). The absorption rate of resveratrol and FUDR in worms during the whole treatment process from high to low was NGM dead, liquid growing, spot dead, the NGM live, and LB medium method. Therefore, the best absorption efficiency of worms was the NGM dead method (*P*<0.05, t-test).

To investigate the relationship between absorption efficiency and drug dosage, we determined the concentration of drugs in worms administrated with the series of dosages for seven days. The drug absorption efficiency at dosage of 50 µM, 100 µM, and 150 µM administrated was 175.83±14.75, 397.20±20.10, and 495.86±19.20 with NGM dead method, and 158.60±20.00, 385.16±9.50, and 492.26±15.25 with liquid growing method, respectively (Table 3).

**Table 3:** The absorption efficiency of worms compared between the NGM dead method and the liquid growing method.

Resveratrol concentration	50 µM	100 µM	150 µM
	test resveratrol concentration (µg/g)	test resveratrol concentration (µg/g)	test resveratrol concentration (µg/g)
**NGM dead method**	175.83±14.75	397.20±20.10	495.86±19.20
**Liquid growing method**	158.60±20.00	385.16±9.50	492.26±15.25

The day 7 worms treated with resveratrol at three concentrations cultured by using NGM dead method and liquid growing method. The table showed the average of three individual experiments for each concentration in each method.

### The metabolism of resveratrol and FUDR in *C. elegans*

**Figure 5:**
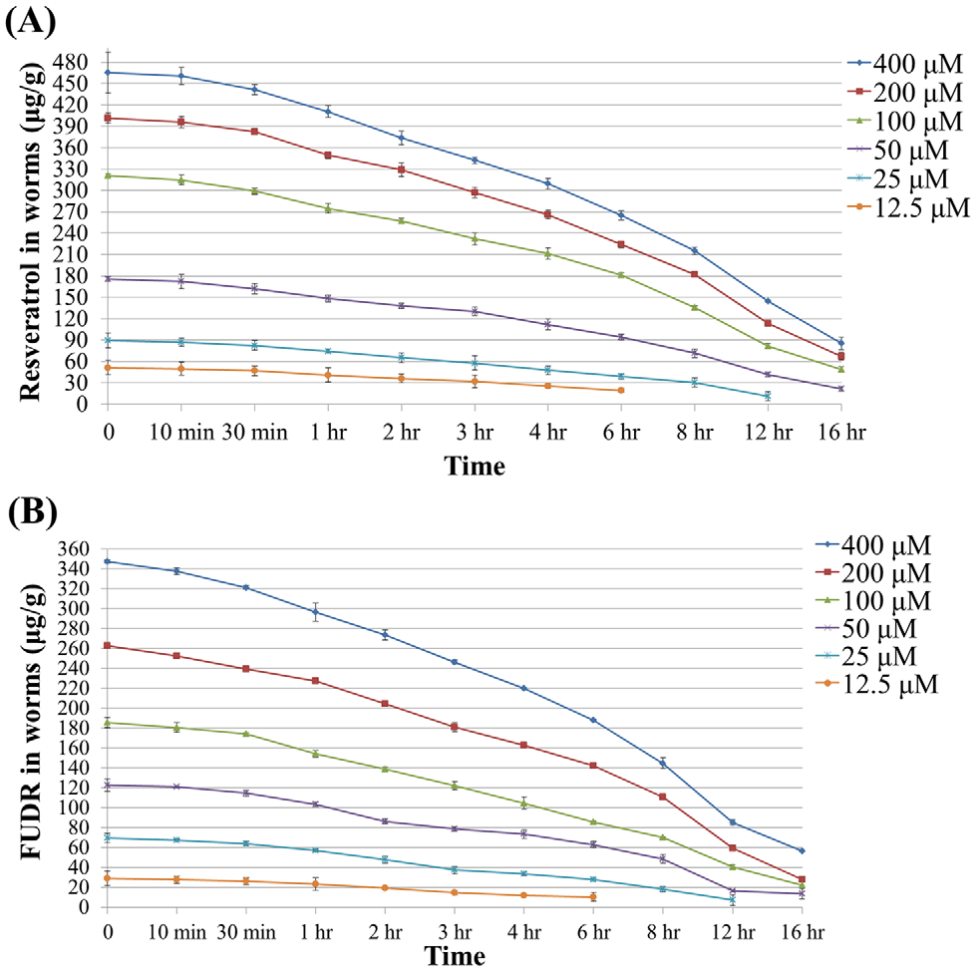
The resveratrol (A) and FUDR (B) catabolism rate inside the worms within 16 hours (µg/g). The worms were cultured by using NGM dead method for 6 hours, then transferred to NGM plates containing no resveratrol or FUDR. The worms were harvested at the 10 min, 30 min, 1 hr, 2 hr, 3 hr, 4 hr, 6 hr, 8 hr, 12 hr and 16 hr after transferring respectively. The details of data were summarized in Tables S3 and S4.

To measure the metabolism of resveratrol and FUDR in *C. elegans*, the concentration of resveratrol and FUDR within *C. elegans* were determined at multiple time points after treatment of drugs with concentrations varied from 400 µM to 12.5 µM for 6 hrs. As indicated in Figure 5, the slopes between the adjacent time interval represent the metabolism rate. The resveratrol and FUDR share the same metabolite pattern (Figure 5, Tables S3 and S4). The metabolism rate increased gradually as time passed on and reached the highest at the time interval of 8 to 12 hours, then decreased followed by the decrease of drug concentration within worms. Generally, the metabolism rate was dose respondent. The higher concentration of drugs has higher metabolism rate at the same time interval.

### Live *E. coli* OP50 lowered the overall drug concentrations in LB medium method and NGM live method

**Figure 6:**
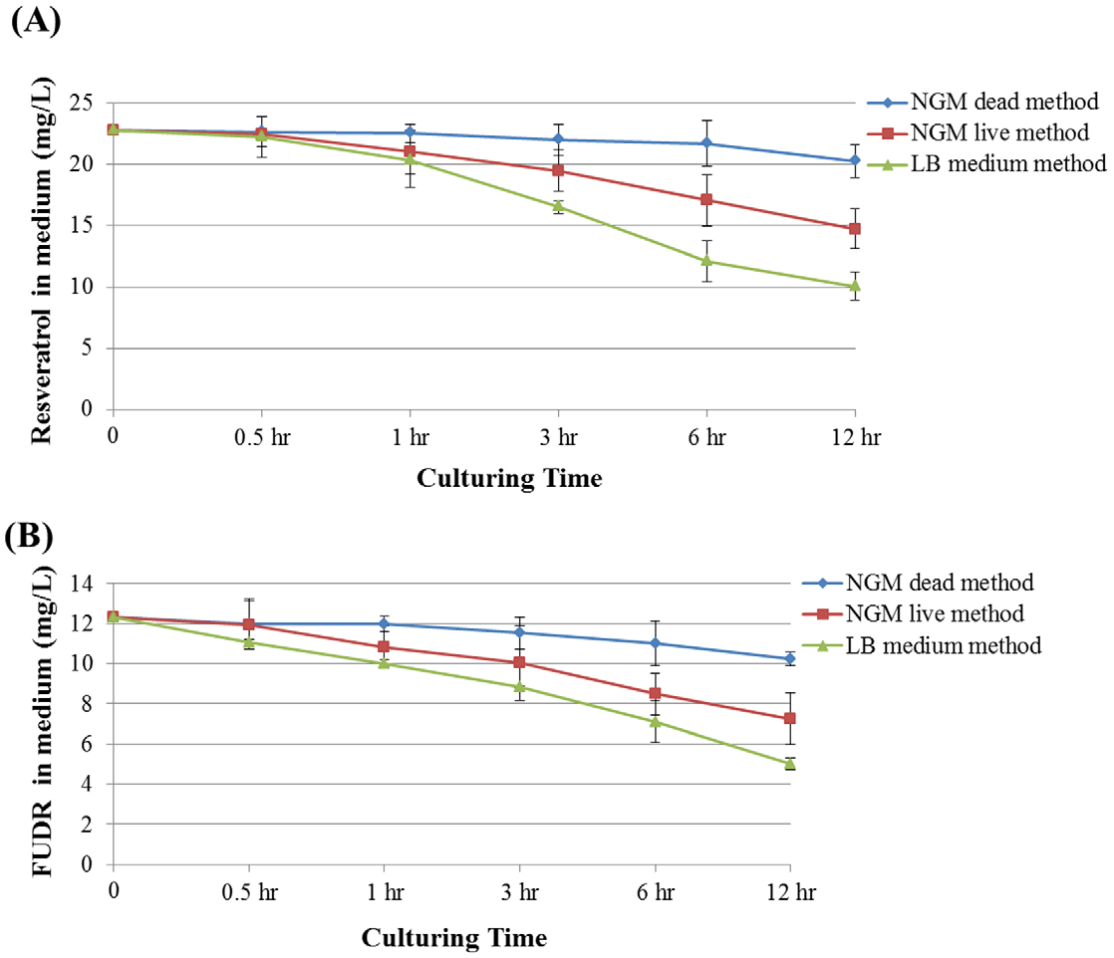
Resveratrol (A) and FUDR (B) concentration in the medium of NGM dead method, NGM live method and LB medium method within 12 hours (mg/L). The initial concentration of resveratrol or FUDR in the medium was 100 µM or 50 µM (0 hr). The medium was crushed and transferred into 15 mL tube at the 0.5 hr, 1 hr, 3 hr, 6 hr and 12 hr after preparing respectively. The same volume of methanol was added into the tube. The mixture was sonicated for 1 hour, then the liquid was collected for HPLC analysis. The details of data were summarized in Table S5.

We suspect that live OP50 *E. coli* would metabolize the drug and lower the overall drug concentrations in the media. To test our hypothesis, we determined the concentrations of resveratrol and FUDR in the medium of LB medium method, NGM live method, and NGM dead method at 0.5 hr, 1 hr, 3 hr, 6 hr and 12 hr after applying live or dead bacteria. The concentration of drug in the medium with dead bacteria degraded slowly over time (Figure 6, Table S5). However, the concentration of drugs in medium with live bacteria incubated for 12 hours were only half of the original concentration. The concentration of drugs in medium with live bacteria decreased much faster than with dead bacteria (Figure 6, Table S5). Above results indicated that the live bacteria could metabolize the drug and lower the drug absorption rate of worms.

## Discussion

As *C. elegans* was more and more widely used in various fields as research and drug screening model, it's important to understand the pharmacological aspect of worms. Here, we established the HPLC method to evaluate the drug concentration in worms administrated by five delivering methods [36]–[42]. Our results showed that about 20,000 worms treated with 100 µM of resveratrol or 50 µM of FUDR could absorb the fraction well beyond the detecting limit of HPLC. The precision (RSD<3.8% for resveratrol and 1.1% for FUDR, Table 2) and repeatability (RSD = 3.8% for resveratrol and 2.5% for FUDR, Table 1) indicate that the HPLC method was sensitive and reliable in detecting the drug absorption efficiency of worms.

The NGM live method [22], [43], [44] and the NGM dead method [30], [45] were widely used by many laboratories in their researches. The drug absorption efficiency of NGM dead was better than NGM live, indicating the live bacteria might have digested part of the drugs. In consistent with this observation, the drug concentration in medium with live bacteria decreased much faster than with dead bacteria. The bacteria live methods performs poorer than bacteria dead methods, indicating worms absorb the drugs mainly from the solution or surface of NGM directly, rather than from the bacteria. For the spot dead method, although the bacteria was dead on the surface of the NGM plates, when the resveratrol or FUDR solution was spotted onto the surface, the solution could immersed into the NGM agar (data not shown), which reduced the availability. This accounted for the drug absorption lower than NGM dead, but higher than NGM live method. Still, spot dead method could reach considerable concentration with less total compounds compared with other drug delivering methods. Both the water-soluble FUDR and resveratrol with low aqueous solubility accumulated within worms steadily and reach the highest concentration within 12–24 hours, then decreased slowly, indicating they share the same absorption pattern in *C. elegans* regardless of delivery methods (Figure 4).

The resveratrol and FUDR share the same metabolite pattern (Figure 5, Tables S3 and S4). The metabolism rate increased gradually as time passed on and reached the highest at the time interval of 8 to 12 hours, then decreased slowly. The decrease of metabolism rate might be caused by the decrease of drug concentration within worms (Figure 5). The higher concentration of drugs has higher metabolism rate at the same time interval. Metabolism rate was dosage responsive and time dependent, indicating the metabolism rate was drug inducible. It would be interesting to investigate the mechanism of the enzyme system responsible for the xenobiotic metabolism.

Although drug administration was common in studies with worms, the concentration of drugs within the worms was unknown, impeding the comparison of the researches between worm and other research models, such as cell culture and animal models. Our results showed that the concentration of resveratrol in worms was from about 300 to 600 mg/kg worms while treated with 100 µM resveratrol, that was comparable with studies conducted on mice, ranged from 4.9 to 400 mg/kg body weight per day resveratrol [46]–[50].

### Conclusion

The resistance of *C. elegans* to pharmacological perturbation appeal an effective approach to make *C. elegans* as a screening tool for novel small bioactive molecules [29]. The HPLC method was effective in determine the concentration of drugs in worms. Resveratrol and FUDR administrated with NGM dead method and liquid growing method achieves the best absorption efficiency in worms. Nevertheless, spot dead method could reach considerable absorption efficiency with much less total amount of drugs. The live bacteria could digest part of the drugs, leading to less drug absorption efficiency. The resveratrol with low aqueous solubility and water soluble FUDR share the same bell-shape accumulation pattern within worms and the same metabolism pattern. The drug concentration within worms was comparable with that in mice, providing a bridge for dose translation from worms to mammals. Our results might help investigators to choose appropriate drug delivering method to worms according to pharmacological dynamics as well as to understand some inconsistent results between studies with different drug delivering methods.

## Supporting Information

Table S1 **The drug absorption efficiency of worms administrated with 100 µM resveratrol by five delivering methods (µg/g).** The concentration of resveratrol in worms was presented as µg/g. The table showed the average of three repeated experiments for each method. - represents the contents of resveratrol were under the limit of detection or not determined (Liquid growing method).^*^ *P*<0.05, ^**^ *P*<0.01, ^***^ *P*<0.001 compared with NGM dead method (t-test).
(DOCX)

Table S2 **The drug absorption efficiency of worms administrated with 50 µM FUDR by five delivering methods (µg/g).** The concentration of FUDR in worms was presented as µg/g. The table showed the average of three repeated experiments for each method. - represents the contents of resveratrol were under the limit of detection or not determined (Liquid growing method).^*^ *P*<0.05, ^**^ *P*<0.01, ^***^ *P*<0.001 compared with NGM dead method (t-test).
(DOCX)

Table S3 **The resveratrol catabolism rate inside the worms within 16 hours (µg/g).** The worms were cultured by using NGM dead method for 6 hours, and then transferred to NGM plates containing no resveratrol. The worms were harvested at the 10 min, 30 min, 1 hr, 2 hr, 3 hr, 4 hr, 6 hr, 8 hr, 12 hr and 16 hr after transferring respectively. - represents the contents of resveratrol were under the limit of detection or not determined.
(DOCX)

Table S4 **The FUDR catabolism rate inside the worms within 16 hours (µg/g).** The worms were cultured by using NGM dead method for 6 hours, and then transferred to NGM plates containing no FUDR. The worms were harvested at the 10 min, 30 min, 1 hr, 2 hr, 3 hr, 4 hr, 6 hr, 8 hr, 12 hr and 16 hr after transferring respectively. - represents the contents of FUDR were under the limit of detection or not determined.
(DOCX)

Table S5 **The drug concentration in the medium of NGM dead method, NGM live method and LB medium method within 12 hours (mg/L).** The initial concentration of resveratrol or FUDR in the medium was 100 µM or 50 µM (0 hr). The medium was crushed and transferred into 15 mL tube at the 0.5 hr, 1 hr, 3 hr, 6 hr and 12 hr after preparing, respectively. The same volume of methanol was added into the tube. The mixture was sonicated for 1 hour, and then the liquid was collected for HPLC analysis.
(DOCX)

## References

[1] Brenner S (1974) The genetics of Caenorhabditis elegans. Genetics 77: 71–94. 4366476 10.1093/genetics/77.1.71 PMC1213120

[2] Artal-Sanz M, de Jong L, Tavernarakis N (2006) Caenorhabditis elegans: a versatile platform for drug discovery. Biotechnol J 1: 1405–1418. 17109493 10.1002/biot.200600176

[3] Burns AR, Kwok TC, Howard A, Houston E, Johanson K, et al (2006) High-throughput screening of small molecules for bioactivity and target identification in Caenorhabditis elegans. Nat Protoc 1: 1906–1914. 17487175 10.1038/nprot.2006.283

[4] Kim S, Paik YK (2008) Developmental and reproductive consequences of prolonged non-aging dauer in Caenorhabditis elegans. Biochem Biophys Res Commun 368: 588–592. 18261976 10.1016/j.bbrc.2008.01.131

[5] Kimble J, Hirsh D (1979) The postembryonic cell lineages of the hermaphrodite and male gonads in Caenorhabditis elegans. Dev Biol 70: 396–417. 478167 10.1016/0012-1606(79)90035-6

[6] Boehm M, Slack F (2005) A developmental timing microRNA and its target regulate life span in C. elegans. Science 310: 1954–1957. 16373574 10.1126/science.1115596

[7] Kniazeva M, Sieber M, McCauley S, Zhang K, Watts JL, et al (2003) Suppression of the ELO-2 FA elongation activity results in alterations of the fatty acid composition and multiple physiological defects, including abnormal ultradian rhythms, in Caenorhabditis elegans. Genetics 163: 159–169. 12586704 10.1093/genetics/163.1.159 PMC1462428

[8] Kniazeva M, Crawford QT, Seiber M, Wang CY, Han M (2004) Monomethyl branched-chain fatty acids play an essential role in Caenorhabditis elegans development. PLoS Biol 2. 10.1371/journal.pbio.0020257 PMC514883 15340492

[9] Zheng J, Greenway FL (2012) Caenorhabditis elegans as a model for obesity research. Int J Obes (Lond) 36: 186–194. 21556043 10.1038/ijo.2011.93

[10] Johnson TE, Wood WB (1982) Genetic analysis of life-span in Caenorhabditis elegans. Proc Natl Acad Sci U S A 79: 6603–6607. 6959141 10.1073/pnas.79.21.6603 PMC347176

[11] Jones DL, Rando TA (2011) Emerging models and paradigms for stem cell ageing. Nat Cell Biol 13: 506–512. 21540846 10.1038/ncb0511-506 PMC3257978

[12] Nass R, Miller DM, Blakely RD (2001) C. elegans: a novel pharmacogenetic model to study Parkinson's disease. Parkinsonism Relat Disord 7: 185–191. 11331185 10.1016/s1353-8020(00)00056-0

[13] Levitan D, Greenwald I (1995) Facilitation of lin-12-mediated signalling by sel-12, a Caenorhabditis elegans S182 Alzheimer's disease gene. Nature 377: 351–354. 7566091 10.1038/377351a0

[14] Dexter PM, Caldwell KA, Caldwell GA (2012) A predictable worm: application of Caenorhabditis elegans for mechanistic investigation of movement disorders. Neurotherapeutics 9: 393–404. 22403010 10.1007/s13311-012-0109-x PMC3337026

[15] Moy TI, Ball AR, Anklesaria Z, Casadei G, Lewis K, et al (2006) Identification of novel antimicrobials using a live-animal infection model. Proc Natl Acad Sci U S A 103: 10414–10419. 16801562 10.1073/pnas.0604055103 PMC1482800

[16] Desalermos A, Muhammed M, Glavis-Bloom J, Mylonakis E (2011) Using C. elegans for antimicrobial drug discovery. Expert Opin Drug Discov 6: 645–652. 21686092 10.1517/17460441.2011.573781 PMC3115622

[17] Anastassopoulou CG, Fuchs BB, Mylonakis E (2011) Caenorhabditis elegans-based model systems for antifungal drug discovery. Curr Pharm Des 17: 1225–1233. 21470110 10.2174/138161211795703753 PMC3719869

[18] Sifri CD, Begun J, Ausubel FM (2005) The worm has turned–microbial virulence modeled in Caenorhabditis elegans. Trends Microbiol 13: 119–127. 15737730 10.1016/j.tim.2005.01.003

[19] Leung MC, Williams PL, Benedetto A, Au C, Helmcke KJ, et al (2008) Caenorhabditis elegans: an emerging model in biomedical and environmental toxicology. Toxicol Sci 106: 5–28. 18566021 10.1093/toxsci/kfn121 PMC2563142

[20] Sellers WR (2011) A blueprint for advancing genetics-based cancer therapy. Cell 147: 26–31. 21962504 10.1016/j.cell.2011.09.016

[21] Onken B, Driscoll M (2010) Metformin induces a dietary restriction-like state and the oxidative stress response to extend C. elegans Healthspan via AMPK, LKB1, and SKN-1. PLoS One 5: e8758. 20090912 10.1371/journal.pone.0008758 PMC2807458

[22] Evason K, Collins JJ, Huang C, Hughes S, Kornfeld K (2008) Valproic acid extends Caenorhabditis elegans lifespan. Aging Cell 7: 305–317. 18248662 10.1111/j.1474-9726.2008.00375.x PMC6838649

[23] Stiernagle T (2006) Maintenance of C. elegans. WormBook 1–11. 10.1895/wormbook.1.101.1 PMC4781397 18050451

[24] Evason K, Huang C, Yamben I, Covey DF, Kornfeld K (2005) Anticonvulsant medications extend worm life-span. Science 307: 258–262. 15653505 10.1126/science.1105299

[25] Petrascheck M, Ye X, Buck LB (2007) An antidepressant that extends lifespan in adult Caenorhabditis elegans. Nature 450: 553–556. 18033297 10.1038/nature05991

[26] Melov S, Ravenscroft J, Malik S, Gill MS, Walker DW, et al (2000) Extension of life-span with superoxide dismutase/catalase mimetics. Science 289: 1567–1569. 10968795 10.1126/science.289.5484.1567

[27] Zarse K, Ristow M (2008) Antidepressants of the serotonin-antagonist type increase body fat and decrease lifespan of adult Caenorhabditis elegans. PLoS One 3: e4062. 19112515 10.1371/journal.pone.0004062 PMC2605556

[28] Keaney M, Gems D (2003) No increase in lifespan in Caenorhabditis elegans upon treatment with the superoxide dismutase mimetic EUK-8. Free Radical Biology and Medicine 34: 277–282. 12521609 10.1016/s0891-5849(02)01290-x

[29] Burns AR, Wallace IM, Wildenhain J, Tyers M, Giaever G, et al (2010) A predictive model for drug bioaccumulation and bioactivity in Caenorhabditis elegans. Nat Chem Biol 6: 549–557. 20512140 10.1038/nchembio.380

[30] Wood JG, Rogina B, Lavu S, Howitz K, Helfand SL, et al (2004) Sirtuin activators mimic caloric restriction and delay ageing in metazoans. Nature 430: 686–689. 15254550 10.1038/nature02789

[31] Guarente L (2005) Calorie restriction and SIR2 genes–towards a mechanism. Mech Ageing De 126: 923–928. 10.1016/j.mad.2005.03.013 15941577

[32] Kaeberlein M, McDonagh T, Heltweg B, Hixon J, Westman EA, et al (2005) Substrate-specific activation of sirtuins by resveratrol. J Biol Chem 280: 17038–17045. 15684413 10.1074/jbc.M500655200

[33] Park SJ, Ahmad F, Philp A, Baar K, Williams T, et al (2012) Resveratrol ameliorates aging-related metabolic phenotypes by inhibiting cAMP phosphodiesterases. Cell 148: 421–433. 22304913 10.1016/j.cell.2012.01.017 PMC3431801

[34] Mitchell DH, Stiles JW, Santelli J, Sanadi DR (1978) Synchronous growth and aging of Caenorhabditis elegans in the presence of fluorodeoxyuridine. J Gerontol 34: 28–36. 10.1093/geronj/34.1.28 153363

[35] Gandhi S, Santelli J, Mitchell DH, Stiles JW, et al (1980) A simple method for maintaining large, aging populations of Caenorhabditis elegans. Mechanisms of Ageing and Development 12 2:137–150. 6445025 10.1016/0047-6374(80)90090-1

[36] Tevell Aberg A, Lofgren H, Bondesson U, Hedeland M (2010) Structural elucidation of N-oxidized clemastine metabolites by liquid chromatography/tandem mass spectrometry and the use of Cunninghamella elegans to facilitate drug metabolite identification. Rapid Commun Mass Spectrom 24: 1447–1456. 20411584 10.1002/rcm.4535

[37] Wypijewska A, Bojarska E, Stepinski J, Jankowska-Anyszka M, Jemielity J, et al (2010) Structural requirements for Caenorhabditis elegans DcpS substrates based on fluorescence and HPLC enzyme kinetic studies. FEBS J 277: 3003–3013. 20546305 10.1111/j.1742-4658.2010.07709.x PMC2907653

[38] Pungaliya C, Srinivasan J, Fox BW, Malik RU, Ludewig AH, et al (2009) A shortcut to identifying small molecule signals that regulate behavior and development in Caenorhabditis elegans. Proc Natl Acad Sci U S A 106: 7708–7713. 19346493 10.1073/pnas.0811918106 PMC2683085

[39] Butcher RA, Fujita M, Schroeder FC, Clardy J (2007) Small-molecule pheromones that control dauer development in Caenorhabditis elegans. Nat Chem Biol 3: 420–422. 17558398 10.1038/nchembio.2007.3

[40] Masuda M, Toriumi C, Santa T, Imai K (2004) Fluorogenic derivatization reagents suitable for isolation and identification of cysteine-containing proteins utilizing high-performance liquid chromatography-tandem mass spectrometry. Anal Chem 76: 728–735. 14750869 10.1021/ac034840i

[41] McColl G, Killilea DW, Hubbard AE, Vantipalli MC, Melov S, et al (2008) Pharmacogenetic analysis of lithium-induced delayed aging in Caenorhabditis elegans. J Biol Chem 283: 350–357. 17959600 10.1074/jbc.M705028200 PMC2739662

[42] Srinivasan J, Kaplan F, Ajredini R, Zachariah C, Alborn HT, et al (2008) A blend of small molecules regulates both mating and development in Caenorhabditis elegans. Nature 454: 1115–1118. 18650807 10.1038/nature07168 PMC2774729

[43] Wilson MA, Shukitt-Hale B, Kalt W, Ingram DK, Joseph JA, et al (2006) Blueberry polyphenols increase lifespan and thermotolerance in Caenorhabditis elegans. Aging Cell 5: 59–68. 16441844 10.1111/j.1474-9726.2006.00192.x PMC1413581

[44] Huang C, Xiong C, Kornfeld K (2004) Measurements of age-related changes of physiological processes that predict lifespan of Caenorhabditis elegans. Proc Natl Acad Sci U S A 101: 8084–8089. 15141086 10.1073/pnas.0400848101 PMC419561

[45] Liao VH, Yu CW, Chu YJ, Li WH, Hsieh YC, et al (2011) Curcumin-mediated lifespan extension in Caenorhabditis elegans. Mech Ageing Dev 132: 480–487. 21855561 10.1016/j.mad.2011.07.008

[46] Ajmo JM, Liang X, Rogers CQ, Pennock B, You M (2008) Resveratrol alleviates alcoholic fatty liver in mice. Am J Physiol Gastrointest Liver Physiol 295: G833–842. 18755807 10.1152/ajpgi.90358.2008 PMC2575919

[47] Barger JL, Kayo T, Vann JM, Arias EB, Wang J, et al (2008) A low dose of dietary resveratrol partially mimics caloric restriction and retards aging parameters in mice. PLoS One 3: e2264. 18523577 10.1371/journal.pone.0002264 PMC2386967

[48] Pearson KJ, Baur JA, Lewis KN, Peshkin L, Price NL, et al (2008) Resveratrol delays age-related deterioration and mimics transcriptional aspects of dietary restriction without extending life span. Cell Metab 8: 157–168. 18599363 10.1016/j.cmet.2008.06.011 PMC2538685

[49] Fonseca-Kelly Z, Nassrallah M, Uribe J, Khan RS, Dine K, et al (2012) Resveratrol neuroprotection in a chronic mouse model of multiple sclerosis. Front Neurol 3: 84. 22654783 10.3389/fneur.2012.00084 PMC3359579

[50] Baur JA, Pearson KJ, Price NL, Jamieson HA, Lerin C, et al (2006) Resveratrol improves health and survival of mice on a high-calorie diet. Nature 444: 337–342. 17086191 10.1038/nature05354 PMC4990206

