# Supplementary material for: Drug Absorption Efficiency in Caenorhbditis elegans Delivered by Different Methods
Source: PLoS One. 2013 Feb 25;8(2):e56877. doi: 10.1371/journal.pone.0056877 (PMC3581574; doi:10.1371/journal.pone.0056877)
Supplement: Table S1 — The drug absorption efficiency of worms administrated with 100 µM resveratrol by five delivering methods (µg/g). The concentration of resveratrol in worms was presented as µg/g. The table showed the average of three repeated experiments for each method. - represents the contents of resveratrol were under the limit of detection or not determined (Liquid growing method).* P<0.05, ** P<0.01, *** P<0.001 compared with NGM dead method (t-test). (DOCX) [file pone.0056877.s001.docx]

**Table S1** The drug absorption efficiency of worms administrated with 100 μM resveratrol by five delivering methods (μg/g).

| Culturing Time | NGM dead method | Liquid growing method | Spot dead method | NGM live method | LB medium method | |
| --- | --- | --- | --- | --- | --- | --- |
| 10 min | 30.28±11.29 | 31.24±8.96 | 25.56±9.48 | 20.75±10.26 | 19.59±3.36 | |
| 30 min | 150.12±2.78 | 160.28±3.36 | 70.09±8.44^***^ | 38.50±1.52^***^ | 21.20±1.09^***^ | |
| 1 hr | 205.26±3.56 | 209.36±8.79 | 75.76±6.30^***^ | 53.95±6.77^***^ | 28.47±1.64^***^ | |
| 3 hr | 281.87±2.14 | 270.59±5.92 | 154.64±14.46^***^ | 92.32±3.11^***^ | 37.18±1.62^***^ | |
| 6 hr | 364.93±3.00 | 340.32±2.89 | 279.56±9.03^***^ | 143.88±5.18^***^ | 86.28±15.62^***^ | |
| 12 hr | 555.48±3.56 | 571.56±5.46 | 476.18±4.17^***^ | 375.14±5.64^***^ | 152.55±8.25*^**^ | |
| 1 day | 593.20 ± 7.35 | 552.60 ± 15.50 | 417.41 ± 20.75^***^ | 352.60 ± 17.50^***^ | 295.86 ± 7.40^***^ | |
| 2 day | 548.78±7.56 | 561.21±4.64 | 376.78±9.58^***^ | 302.28±8.96^***^ | 282.55±9.65^***^ | |
| 4 day | 464.53±12.36 | 449.32±20.29 | 300.25±8.31^***^ | 273.56±18.86^***^ | 268.28±18.35^***^ | |
| 7 day | 393.93 ± 3.52 | 385.16 ± 9.50 | 296.86 ± 2.69^**^ | 263.90 ± 5.95^**^ | 242.00 ± 21.90^***^ | |
| 14 day | 117.00 ± 5.30 | - | - | - | - |  |
| 20 day | 19.06 ± 8.50 | - | - | - | - |  |

The concentration of resveratrol in worms was presented as μg/g. The table showed the average of three repeated experiments for each method. - represents the contents of resveratrol were under the limit of detection or not determined (Liquid growing method).^*^*P*<0.05, ^**^*P*<0.01, ^***^*P*< 0.001 compared with NGM dead method (t-test).
