# Supplementary material for: Drug Absorption Efficiency in Caenorhbditis elegans Delivered by Different Methods
Source: PLoS One. 2013 Feb 25;8(2):e56877. doi: 10.1371/journal.pone.0056877 (PMC3581574; doi:10.1371/journal.pone.0056877)
Supplement: Table S2 — The drug absorption efficiency of worms administrated with 50 µM FUDR by five delivering methods (µg/g). The concentration of FUDR in worms was presented as µg/g. The table showed the average of three repeated experiments for each method. - represents the contents of resveratrol were under the limit of detection or not determined (Liquid growing method).* P<0.05, ** P<0.01, *** P<0.001 compared with NGM dead method (t-test). (DOCX) [file pone.0056877.s002.docx]

**Table S2** The drug absorption efficiency of worms administrated with 50 μM FUDR by five delivering methods (μg/g).

| Culturing Time | NGM dead method | Liquid growing method | Spot dead method | NGM live method | LB medium method |
| --- | --- | --- | --- | --- | --- |
| 10 min | 18.34±8.39 | 17.59±6.35 | 18.56±5.37 | 19.65±2.13 | 14.26±8.73 |
| 30 min | 63.42±0.12 | 61.14±1.32 | 58.82±0.14^**^ | 55.92±0.28^***^ | 53.64±0.38^***^ |
| 1 hr | 96.32±0.60 | 101.58±0.58 | 63.18±0.16^***^ | 73.02±0.50^***^ | 68.53±0.50^***^ |
| 3 hr | 116.3±0.69 | 112.53±2.64 | 110.43±0.18^***^ | 81.31±0.23^***^ | 75.58±0.3^***^ |
| 6 hr | 129.86±2.46 | 132.28±0.69 | 114.52±0.20^**^ | 81.42±0.34^***^ | 77.06±0.07^***^ |
| 12 hr | 174.65±1.30 | 169.69±2.38 | 129.92±0.77^***^ | 87.28±1.22^***^ | 92.93±1.82^***^ |
| 1 day | 180.37±0.88 | 178.20±12.55 | 163.32±2.12^***^ | 89.28±0.12^***^ | 85.26±1.53^***^ |
| 2 day | 170.75±11.33 | 160.65±9.89 | 139.82±3.28^***^ | 85.55±7.52^***^ | 76.73±11.35^***^ |
| 4 day | 139.86±7.69 | 132.89±10.02 | 124.32±3.59^**^ | 79.46±5.37^***^ | 71.69±8.65^***^ |
| 7 day | 127.08±20.44 | 120.00±8.56 | 109.02±0.42^***^ | 75.98±1.73^***^ | 61.48±3.98^***^ |
| 14 day | 106.94±0.36 | 103.24±5.36 | 85.48±0.12^***^ | 67.68±0.94^***^ | 44.68±0.64^***^ |

The concentration of FUDR in worms was presented as μg/g. The table showed the average of three repeated experiments for each method. - represents the contents of resveratrol were under the limit of detection or not determined (Liquid growing method).^*^*P*<0.05, ^**^*P*<0.01, ^***^*P*< 0.001 compared with NGM dead method (t-test).
