# Supplementary material for: Drug Absorption Efficiency in Caenorhbditis elegans Delivered by Different Methods
Source: PLoS One. 2013 Feb 25;8(2):e56877. doi: 10.1371/journal.pone.0056877 (PMC3581574; doi:10.1371/journal.pone.0056877)
Supplement: Table S3 — The resveratrol catabolism rate inside the worms within 16 hours (µg/g). The worms were cultured by using NGM dead method for 6 hours, and then transferred to NGM plates containing no resveratrol. The worms were harvested at the 10 min, 30 min, 1 hr, 2 hr, 3 hr, 4 hr, 6 hr, 8 hr, 12 hr and 16 hr after transferring respectively. - represents the contents of resveratrol were under the limit of detection or not determined. (DOCX) [file pone.0056877.s003.docx]

**Table S3** The resveratrol catabolism rate inside the worms within 16 hours (μg/g).

|  | 400 (μM) | 200 (μM) | 100 (μM) | 50 (μM) | 25 (μM) | 12.5 (μM) |
| --- | --- | --- | --- | --- | --- | --- |
| 0 min | 465.35±28.73 | 401.61±7.25 | 321.24±2.15 | 175.82±3.31 | 89.65±10.25 | 51.36±9.83 |
| 10 min | 460.64(98.99)±12.36 | 395.82(98.56)±8.15 | 314.94(98.04)±7.02 | 172.08(97.87)±10.36 | 87.00(97.04)±5.51 | 49.58(96.53)±9.14 |
| 30 min | 441.20(94.81) ±7.10 | 382.57(95.26) ±4.12 | 299.01(93.08)±4.04 | 162.35(92.34) ±7.13 | 82.48(92.01)±6.93 | 47.03(91.57) ±6.70 |
| 1 hr | 410.67(88.25)±8.41 | 349.36(86.99)±4.47 | 274.85(85.56)±6.62 | 148.36(84.38)±4.64 | 74.47(83.07) ±2.76 | 41.08(80.00)±10.21 |
| 2 hr | 373.35(80.23)±9.52 | 328.92(81.90) ±9.63 | 256.90(79.97)±4.10 | 137.98(78.48) ±4.05 | 65.11(72.63)±6.67 | 36.24(70.58) ±6.03 |
| 3 hr | 342.4(73.58) ±4.38 | 296.95(73.94) ±7.36 | 232.03(72.23)±8.25 | 130.46(74.20)±5.69 | 57.84(64.52) ±10.23 | 31.98(62.27)±8.95 |
| 4 hr | 309.41(66.49)±7.63 | 266.18(66.28) ±6.14 | 211.66(65.89)±8.18 | 112.17(63.80) ±7.29 | 47.54(53.03)±5.68 | 25.78(50.19) ±3.25 |
| 6 hr | 265.2(56.99)±6.32 | 224.26(55.84)±4.35 | 181.21(56.41)±3.06 | 94.32(53.65)±3.79 | 38.89(43.39) ±3.34 | 19.44(37.85)±2.59 |
| 8 hr | 215.27(46.26)±5.35 | 181.96(45.31) ±3.70 | 135.72(42.25)±2.59 | 71.66(40.76) ±6.16 | 30.37(33.88)±6.04 | - |
| 12 hr | 144.90(31.14)±1.33 | 113.5(28.26)±4.19 | 81.37(25.33)±4.16 | 41.84(23.80)±3.20 | 11.07(12.35)±6.15 | - |
| 16 hr | 85.39(18.35)±8.65 | 67.22(16.74)±5.06 | 48.54(15.11)±4.12 | 21.42(12.18)±3.35 | - | - |

The worms were cultured by using NGM dead method for 6 hours, and then transferred to NGM plates containing no resveratrol. The worms were harvested at the 10 min, 30 min, 1 hr, 2 hr, 3 hr, 4 hr, 6 hr, 8 hr, 12 hr and 16 hr after transferring respectively. - represents the contents of resveratrol were under the limit of detection or not determined.
