# Supplementary material for: Drug Absorption Efficiency in Caenorhbditis elegans Delivered by Different Methods
Source: PLoS One. 2013 Feb 25;8(2):e56877. doi: 10.1371/journal.pone.0056877 (PMC3581574; doi:10.1371/journal.pone.0056877)
Supplement: Table S4 — The FUDR catabolism rate inside the worms within 16 hours (µg/g). The worms were cultured by using NGM dead method for 6 hours, and then transferred to NGM plates containing no FUDR. The worms were harvested at the 10 min, 30 min, 1 hr, 2 hr, 3 hr, 4 hr, 6 hr, 8 hr, 12 hr and 16 hr after transferring respectively. - represents the contents of FUDR were under the limit of detection or not determined. (DOCX) [file pone.0056877.s004.docx]

**Table S4** The FUDR catabolism rate inside the worms within 16 hours (μg/g).

|  | 400 (μM) | 200 (μM) | 100 (μM) | 50 (μM) | 25 (μM) | 12.5 (μM) |
| --- | --- | --- | --- | --- | --- | --- |
| 0 min | 347.45±1.79 | 262.71±2.38 | 185.74±5.39 | 122.82±6.31 | 69.65±4.38 | 29.16±7.26 |
| 10 min | 337.93(97.26)±3.40 | 252.71(96.19)±3.15 | 180.80(97.34)±5.02 | 120.95(98.48)±0.99 | 67.66(97.14)±1.05 | 27.86(95.53)±3.68 |
| 30 min | 321.54(92.54) ±2.10 | 239.43(91.13) ±1.03 | 174.59(93.95)±1.01 | 114.64(93.34) ±3.09 | 63.94(91.80)±2.31 | 26.28(90.12) ±3.35 |
| 1 h | 296.65(85.38)±8.91 | 227.42(86.57)±1.14 | 154.34(83.10)±3.31 | 103.54(84.30)±2.37 | 57.09(81.97) ±1.38 | 23.36(80.12)±6.38 |
| 2 h | 273.99(78.86)±5.21 | 204.65(77.90) ±1.24 | 139.06(74.87)±2.05 | 86.56(70.48) ±2.51 | 47.82(68.63)±3.33 | 19.12(65.58) ±1.02 |
| 3 h | 246.62(70.98) ±2.22 | 181.11(68.94) ±4.39 | 122.40(65.9)±4.13 | 78.85(64.20)±2.04 | 37.56(53.92) ±3.66 | 14.84(50.90)±2.16 |
| 4 h | 219.90(63.29)±0.62 | 162.82(61.98) ±3.07 | 104.76(56.04)±6.07 | 73.44(59.80) ±4.04 | 33.69(48.37)±1.68 | 12.03(41.26) ±0.88 |
| 6 h | 188.14(54.14)±0.86 | 142.11(54.09)±2.17 | 85.52(46.04)±1.45 | 63.12(51.39)±2.87 | 28.18(40.46) ±1.17 | 10.45(35.85)±4.13 |
| 8 h | 144.88(41.7)±5.35 | 111.12(42.3) ±1.85 | 70.28(37.84)±0.49 | 48.83(39.76) ±4.06 | 18.23(26.17)±3.02 | - |
| 12 h | 85.26(24.54)±2.66 | 59.44(22.62)±1.89 | 40.64(21.88)±2.08 | 16.69(20.51)±1.09 | 7.12(10.22)±5.32 | - |
| 16 h | 56.82(16.35)±0.96 | 28.17(10.74)±2.53 | 22.49(12.11)±3.06 | 13.73(11.18)±5.09- | | - |

The worms were cultured by using NGM dead method for 6 hours, and then transferred to NGM plates containing no FUDR. The worms were harvested at the 10 min, 30 min, 1 hr, 2 hr, 3 hr, 4 hr, 6 hr, 8 hr, 12 hr and 16 hr after transferring respectively. - represents the contents of FUDR were under the limit of detection or not determined.
