# Supplementary material for: Drug Absorption Efficiency in Caenorhbditis elegans Delivered by Different Methods
Source: PLoS One. 2013 Feb 25;8(2):e56877. doi: 10.1371/journal.pone.0056877 (PMC3581574; doi:10.1371/journal.pone.0056877)
Supplement: Table S5 — The drug concentration in the medium of NGM dead method, NGM live method and LB medium method within 12 hours (mg/L). The initial concentration of resveratrol or FUDR in the medium was 100 µM or 50 µM (0 hr). The medium was crushed and transferred into 15 mL tube at the 0.5 hr, 1 hr, 3 hr, 6 hr and 12 hr after preparing, respectively. The same volume of methanol was added into the tube. The mixture was sonicated for 1 hour, and then the liquid was collected for HPLC analysis. (DOCX) [file pone.0056877.s005.docx]

**Table S5** The drug concentration in the medium of NGM dead method, NGM live method and LB medium method within 12 hours (mg/L).

|  |  | 0 hr | 0.5hr | 1hr | 3hr | 6hr | 12hr |
| --- | --- | --- | --- | --- | --- | --- | --- |
| NGM dead method | **Resveratrol** | 22.82 | 22.68±1.21 | 22.55±0.76 | 22.03±1.25 | 21.74±1.89 | 20.29±1.35 |
|  | **FUDR** | 12.31 | 12.00±1.26 | 11.98.±0.39 | 11.54±0.79 | 11.02±1.11 | 10.26±0.34 |
| NGM live method | **Resveratrol** | 22.82 | 22.51±0.33 | 21.03±1.82 | 19.5±1.72 | 17.08±2.11 | 14.75±1.65 |
|  | **FUDR** | 12.31 | 11.95±1.22 | 10.82±1.05 | 10.03±1.87 | 8.48±1.02 | 7.25±1.28 |
| LB medium method | **Resveratrol** | 22.82 | 22.24±1.66 | 20.34±2.18 | 16.53±0.52 | 12.08±1.66 | 10.08±1.13 |
|  | **FUDR** | 12.31 | 11.05±0.18 | 10.02±0.16 | 8.84±0.04 | 7.12±1.04 | 5.02±0.29 |

The initial concentration of resveratrol or FUDR in the medium was100 μM or 50μM (0 hr).The medium was crushed and transferred into 15 mL tube at the 0.5 hr, 1 hr, 3 hr, 6 hr and 12 hr after preparing, respectively. The same volume of methanol was added into the tube. The mixture was sonicated for 1 hour, and then the liquid was collected for HPLC analysis.
